# Chemical and mineralogical characteristics of tropical soils on the transport of Na^+^, K^+^, Ca^2+^, and Mg^2+^ from the application of treated wastewater

**DOI:** 10.1002/jeq2.70105

**Published:** 2025-10-23

**Authors:** Marina N. Merlo, Michael S. Thebaldi, Miguel A. C. Alvarez, Daniela C. de Jesus, Jaqueline dos S. Soares, Elvis M. de C. Lima, Mateus A. da Silva, Luiz A. Lima, Luiz F. C. de Oliveira

**Affiliations:** ^1^ Department of Water Resources Universidade Federal de Lavras Lavras Minas Gerais Brazil; ^2^ Department of Agricultural Engineering Universidad Nacional de Agricultura Catacamas Olancho Honduras

## Abstract

With the search for water security, the reuse of water in irrigation becomes interesting and inevitable. Given the complexity of soil–solute interactions, the use of simplified artificial solutions represents an innovative approach, as it facilitates understanding these interactions while posing no risk to human health or the environment. However, the more complex and inferior the quality of the water, the greater the environmental contamination risk. The objective of this study was to investigate ion retention in three tropical soils (Oxisol, Inceptisol, and Entisol Quartzipsamment) by applying treated domestic wastewater (TWW) and artificial wastewater, both with the same concentrations of Na^+^, K^+^, Ca^2+^, and Mg^2+^ as the TWW. Physical, chemical, and mineralogical properties of the soil were determined. Multivariate analysis was performed to elucidate the relationships between the solutes’ transport parameters, the soils’ mineralogy and texture, and the variation in their chemical characteristics resulting from wastewater application. Oxisol and Entisol Quartzipsamment contributed to principal component 1. The second principal component had a negative contribution from Inceptisol. Oxisol and Entisol Quartzipsamment showed the longest delays of ion transport relative to the wetting front. Inceptisol had the lowest retardation factors and the lowest affinity for ions. For the TWW on Oxisol, Na^+^ was the first ion to reach a relative concentration equal to 1, at an average pore volume of 4.3; however, the last pore volume collected for Ca^2+^ was 12.3, with a relative concentration of 0.6, showing that sodium in TWW is a point of attention, as observed by the low affinity with soils.

## Core Ideas

- The evaluated wastewater quality resembles diluted wastewater.
- The Na^+^ is a point of attention due to its high mobility in tropical soils.
- The wastewater increased the pH and availability of K^+^, Ca^2+^, and Mg^2+^ in tropical soils.
- The artificial wastewater can be used to predict the treated domestic wastewater ions transport behavior, except for the Ca^2+^ retardation factor.

## Plain Language Summary

The use of wastewater for irrigation of crops is interesting, since it allows the recycling of nutrients for plant development. But, if the application of this wastewater to the soil is not performed correctly, the ions present in the wastewater can move through the soil, and cause contamination of surface water and groundwater. We evaluated the transport of some ions in tropical soils, relating it to their chemical and mineralogical characteristics, so we could understand which factors would be most important to ensure safe wastewater application to the soils. Even with wastewater with relatively good quality, the Na was a key point of attention regarding soil and water contamination. The application of sewage to the soil also led to enhanced availability of K^+^, Ca^2+^, and Mg^2+^, a fact of relevant interest from an agricultural perspective, as these nutrients are essential for good plant development and contribute to the soil being suitable for its development.

**Abbreviations**
AWW: artificial wastewater
CEC: cation exchange capacity
PC1: first principal component
PC2: second principal component
Pe: Péclet number
Sb: sum of exchangeable bases
TWW: treated domestic wastewater

## 1 INTRODUCTION

The challenge in conciliating water availability with the growing food demand is substantial, primarily due to the impacts of climate change and environmental pollution. Water consumption has increased significantly, and in the future, water supply may be further compromised due to this imbalance (Garnier et al., 2015).

Agriculture is the largest user of water worldwide, and the decreasing availability of water has emphasized the need for alternative sources, such as the reuse of treated wastewater for irrigation, which contains essential nutrients for crop growth (Lavrnić et al., 2017). According to Sustainable Development Goal 6, to ensure water availability, sustainable resource management, and sanitation, untreated wastewater must be reduced by 50% by 2030, promoting nutrient cycling and the safe reuse of treated wastewater (Vanham et al., 2018).

Based on data from the National Sanitation Information System—SINISA (SINISA, 2024), for the year 2023, 59.7% of the Brazilian population had access to a sewage collection network, and 62.3% of the total sewage volume generated was collected. In terms of treated sewage, 78.9% of the collected volume received treatment, but this represents only 49.0% of the total volume (SINISA, 2024). The wastewater degree of treatment is influenced by its specific characteristics, the self‐purification capacity of the receiving water body, and the properties of the wastewater itself (SINISA, 2024).

The use of treated wastewater for fertigation can provide both economic and environmental benefits (Coelho et al., 2020). Among these benefits are increased soil fertility, a reduced water footprint in agriculture (Barbosa et al., 2022), and a decrease in the potential for environmental contamination from wastewater when it is directly discharged into receiving water bodies (Coelho et al., 2020).

However, to ensure the safe use of wastewater, it is essential to monitor its quality to prevent soil degradation and the contamination of plants and groundwater. In addition, the physical, chemical, and hydraulic properties of soils are crucial for the safe use of treated wastewater in agriculture. However, each type of soil may exhibit distinct behavior regarding ion movement and retention. This variability must be carefully investigated, as several factors influence the process. These factors include soil mineralogy, cation exchange capacity (CEC), affinity between adsorption sites and the ions, the size of the ions’ hydrated radius, as well as the ions' charge and concentration. Each of these factors contributes to the complexity of soil‐ion interactions, and understanding them is essential. Oxisol is a typical example of a soil with unique properties: even when it has a clayey texture, its water retention properties resemble sandy soils, such as Entisol Quartzipsamment (Van Wambeke et al., 1983), resulting in high soil saturated hydraulic conductivity (Nogueira et al., 2021) and could present a risk for groundwater contamination. In addition, highly weathered soils are abundant in kaolinite clay, iron, and aluminum, which can displace and leach potassium, calcium, and magnesium (Paramisparam et al., 2021). This leaching process occurs in tropical regions due to high temperatures and heavy rainfall, which removes the previously mentioned non‐acidic cations and replaces them with Al^3+^, H^+^, and Fe^2+^ at the adsorption sites (Ng et al., 2022). This ion substitution leads to the acidification of tropical soils, which reduces the availability of K^+^, Ca^2+^, and Mg^2+^, reducing crop productivity (Fageria & Nascente, 2014; Malavolta et al., 1997).

Many studies focus on soil salinization resulting from the application of treated wastewater (Elfanssi et al., 2018; Hashem & Qi, 2021; Jahany & Rezapour, 2020; Ofori et al., 2021); however, the type of treatment used and the wastewater quality (Gao et al., 2021; Mendes Reis et al., 2021; Mishra et al., 2023) can safely provide benefits to crops and the environment.

Gao et al. (2021) performed a meta‐analysis of 21 articles and verified that the application of treated wastewater promoted the accumulation of sodium, potassium, calcium, and magnesium, especially in sandy soil compared to clay soil. Shilpi et al. (2018) also verified an increase in Na^+^, Ca^2+^, and Mg^2+^ contents in sandy clay loam soil irrigated with various types of wastewaters (treated municipal, winery, dairy, and abattoir) and dilutions.

Other studies have evaluated the impacts that irrigation with treated wastewater could have on crop development, groundwater, and society, emphasizing that the use of this water source brings benefits in relation to fresh water (Abdelrahman et al., 2011; Alkhamisi et al., 2011; Freihat et al., 2021; Gao et al., 2021; Gupta et al., 1998).

In a study by Mendes Reis et al. (2021), millet was cultivated using treated domestic wastewater (TWW) for irrigation at five different dilution levels in both clayey and loamy‐sandy soils. The authors reported that increasing the percentage of TWW in the irrigation water has a positive impact on millet dry matter production and nutrient uptake. This beneficial effect was attributed to an observed increase in CEC, available nutrients, and base saturation in both soil types. However, they noted that applying 100% TWW to the clayey soil resulted in a reduction of root dry matter compared to the 75% treatment, which could be associated with increased soil salinization and sodification.

Elfanssi et al. (2018) assessed the effects on soil properties and alfalfa growth in sandy soil due to the application of raw domestic wastewater, TWW, and well water. Both raw and treated wastewater contain significantly higher concentrations of exchangeable cations, specifically Na, K, Ca, and Mg, when compared to well water. During the alfalfa growing seasons, the application of raw domestic wastewater increased soil salinity, indicating its inferior quality compared to TWW and well water, which exhibited lower salinity.

El Moussaoui et al. (2019) found an increase in yield and macro‐ and micronutrient concentrations in alfalfa irrigated with treated and raw domestic wastewater, in addition to increasing productivity and reducing the cost of mineral fertilization. Mohammad Rusan et al. (2007) concluded that productivity and fertility can be improved with the use of wastewater; however, irrigation management practices are recommended, as well as monitoring of soil quality due to the addition of salts, nutrients, and metals above the levels tolerated by crops. Ungureanu et al. (2020) present that the use of treated wastewater can conserve available water resources and increase the population's food security.

In this regard, the study of the soil‐ion interaction allows determining the dispersion, mobility, and velocity of advance of a contaminant in relation to the wetting front that advances through the porous media, in addition to quantifying the adsorption capacity of the soil, aiming at environmental preservation. It must be stressed that there is a delicate balance between the benefits and risks of applying treated wastewater to soil, particularly in tropical soils, which cover a significant part of the world. These soils naturally exhibit low fertility and are mostly found in areas with higher rainfall intensity, a fact that underscores the relevance of this work.

Based on the foundational knowledge of soil physics, chemistry, and hydrology, sandy soils are more prone to lower cation and water retention capacities. This implies a higher risk of leaching in areas where these soils are predominant. The opposite is expected in areas primarily composed of soils with higher CEC. However, due to the diversity and variability of soil attributes across different regions of the globe, investigating the nuances of these interactions is crucial. Although the tendency for soils' exchange complexes to retain higher valence cations is expected, wastewaters' complex quality originates from their diversity of ions, presence of organic matter and organic nutrients, detergents, inorganic solids, and microorganisms, among others. Thus, determining whether the expected transport behavior of certain target ions can be evaluated using simpler artificial solutions is innovative, technically and scientifically relevant, because it can aid in understanding the complex interactions between soil and solution characteristics in this scenario, without the risk of human and environmental contamination.

Thus, the objective of this study was to investigate the transport of Na^+^, K^+^, Ca^2+^, and Mg^2+^ ions from TWW in three classes of tropical soils with different, and even unique, physical, chemical, and hydraulic characteristics, such as mineralogical composition, fertility, texture, hydraulic conductivity, and affinity with ions, to assess potential groundwater contamination risks and support its safe application in agriculture.

**Core Ideas**

- The evaluated wastewater quality resembles diluted wastewater.
- The Na^+^ is a point of attention due to its high mobility in tropical soils.
- The wastewater increased the pH and availability of K^+^, Ca^2+^, and Mg^2+^ in tropical soils.
- The artificial wastewater can be used to predict the treated domestic wastewater ions transport behavior, except for the Ca^2+^ retardation factor.

## 2 MATERIALS AND METHODS

### 2.1 Wastewater and soil characterization

The miscible displacement tests were carried out at the Department of Water Resources of the Universidade Federal de Lavras (UFLA). Two types of wastewater were used: TWW from the Sewage Treatment Plant of UFLA (STP—UFLA) and artificial wastewater (AWW) containing Na^+^, K^+^, Ca^2+^, and Mg^2+^ ions at the same concentration as those in the TWW from the STP—UFLA. The similarity between TWW and AWW lies only in their identical concentration of the target ions (Na^+^, K^+^, Ca^2+^, and Mg^2+^). Consequently, it is relevant to evaluate if the application of the less complex solution can be used to predict the transport behavior of the target ions examined in this work.

The AWW was composed of salts of NaCl P.A. A.C.S. (minimum content of 99%), KCl P.A. A.C.S. (content of 99%–100.5%), CaCl_2_ P.A. (content of 99%–107%), and MgCl_2_ P.A. (content of 99%–102%). The pH of the AWW was 5.65.

The soils used were an Oxisol and Inceptisol, which were collected at the university campus in Lavras, MG/Brazil, at the coordinates 21°13′41″ S 44°58′18″ W and 21°13′48″ S 44°59′12″ W, respectively, and an Entisol Quartzipsamment, which was collected in Itumirim, MG/Brazil (21°21′40″ S 44°52′22″ W). These soils are named, respectively, as Latossolo Vermelho, Cambissolo Háplico, and Neossolo Quartzarênico, according to the Brazilian Soil System Classification (Santos et al., 2018).

The TWW was collected in a downstream section of STP‐UFLA after passing by a coarse and fine grating, grease separator, flow distribution box, upflow anaerobic sludge blanket unit, fixed‐film activated sludge, and sand filter. The sampling was performed manually, only once, in 50‐L plastic containers.

During the experimental period, the following wastewater quality variables (Table 1) were evaluated, according to the methodology presented in APHA, AWWA and WEF (2022): sodium, potassium, calcium, and magnesium, by flame photometry and atomic absorption spectrometry; pH and electrical conductivity, by potentiometry; total, fixed, and volatile solids, by gravimetry; turbidity, by nephelometry; and chemical oxygen demand, by the colorimetric method.

**TABLE 1:** Quality variables of treated wastewater from Sewage Treatment Plant (STP)‐Universidade Federal de Lavras (UFLA).

Variables	Output STP‐UFLA	Variables	Output STP‐UFLA
Sodium, mg L^−1^	22.6	Total solids, mg L^−1^	298.0
Potassium, mg L^−1^	9.5	Fixed solids, mg L^−1^	123.0
Calcium, mg L^−1^	4.0	Volatile solids, mg L^−1^	175.0
Magnesium, mg L^−1^	6.2	Turbidity, NTU	8.6
pH	7.51	Chemical oxygen demand, mg L^−1^	88.9
Electrical conductivity, dS m^−1^	0.498	Sodium adsorption ratio (SAR; meq/L)^0.5^	10.0

The TWW sample was filtered to remove solids, refrigerated, stored at 0°C, and used daily according to the requirements of the miscible displacement experiment.

The deformed soil samples were collected from a depth of 0–0.40 m, from areas not submitted to agricultural activity. To this end, all organic material was removed from the soil, and four simple samples were collected (Arruda et al., 2014). These samples were mixed, dried at room temperature, disaggregated, homogenized, mechanically quartered, and mechanically sieved in a 0.002 m mesh.

**FIGURE 1:**
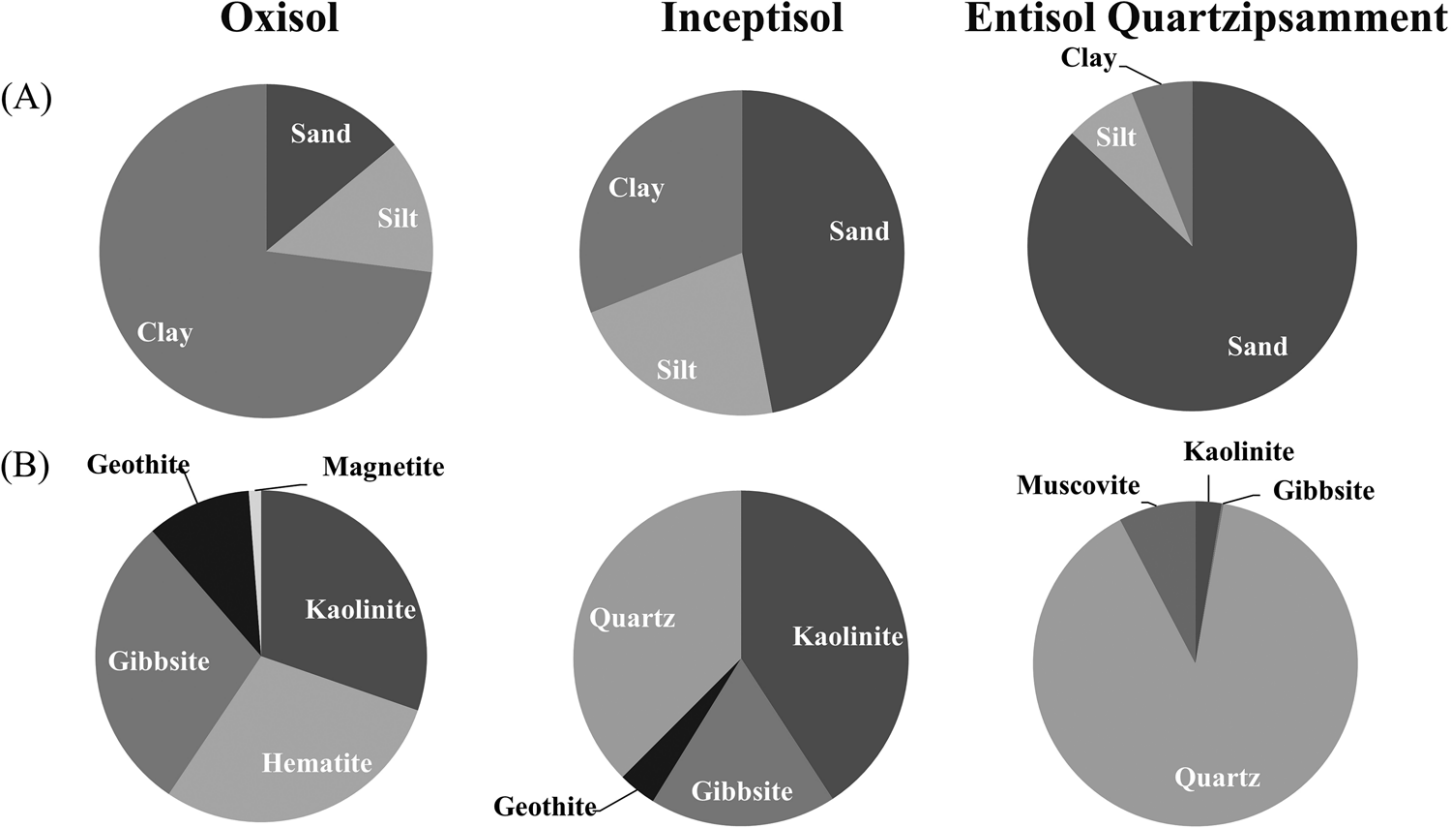
Textural classification of the soils studied, according to Santos et al. (2013)(A), and minerals present from the crystal phases of the soils (B). *Source*: CPMTC—UFMG (2024).

The soils were characterized by their physical, mineralogical, and chemical properties (Teixeira et al., 2017). The particle size distribution was determined using the pipette method (Figure 1A). For the mineralogical characterization, crystalline phase identification by X‐ray diffraction was used to examine the clay and silt fractions of the soils, identifying the minerals and quantifying their proportions (Figure 1B).

The average soil particle density was determined by the volumetric balloon method, calculated by Equation (1).

(1) $$\rho_{p}=\frac{m_{\mathrm{ds}}}{V_{s}}$$

where *ρ*_p_ is the soil particle density (g cm^−3^), *m*_ds_ is the mass of dry soil at 105°C (g), and *V*_s_ is the volume occupied by the soil inside the balloon (cm^3^). The particle density values obtained were 2.61 g cm^−3^ for Oxisol, 2.51 g cm^−3^ for Inceptisol, and 2.59 g cm^−3^ for Entisol Quartzipsamment.

To determine the soil bulk density, literature was consulted for in‐field total porosity values of the soil classes studied at a depth of 0–0.40 m, and an average value was calculated. Thus, the porosity for the Oxisol was 0.61 cm^3^ cm^−3^ (Beutler et al., 2002; Silva & Castro, 2015; Silva et al., 2009), for the Inceptisol was 0.55 cm^3^ cm^−3^ (Oliveira et al., 2021; Portugal et al., 2008), and for the Entisol Quartzipsamment was 0.36 cm^3^ cm^−3^ (Ramos et al., 2020). The soil bulk density was calculated by Equation (2).

(2) $$\rho_{s}=\rho_{p}\left(1-\alpha \right)$$

where *ρ*_s_ is the soil bulk density (g cm^−3^) and *α* is the soil total porosity (cm^3^ pores cm^−^^3^ soil). The calculated soil bulk densities were 1.01 g cm^−3^ for Oxisol, 1.12 g cm^−3^ for Inceptisol, and 1.65 g cm^−3^ for Entisol Quartzipsamment.

With this, the soil mass to fill the columns was determined (Equation 3).

(3) $$M_{s}=\rho_{s}.V$$

where *M*_s_ is the soil mass in the column (g) and *V* is the volume of the column (cm^3^).

By Equation (3), and considering the moisture of the air‐dried soil, the mass of dry soil to be added to the columns was determined. The error admitted in this process was 3%. The columns were constructed from rigid PVC with an inner diameter of 0.044 m and a height of 0.20 m. At the lower end of the cylinders, segments of permeable fabric and elastic gum were placed to hold the soil. The soil columns were assembled to standardize soil bulk density, ensuring that variations in this property would not significantly influence the results obtained. Soil was added to the column in layers, each one compacted using a rubber weight with a diameter slightly smaller than that of the PVC column.

Regarding the soils chemical characterization, the following variables were analyzed: organic carbon, pH in water, phosphorus, potassium, calcium, magnesium, aluminum, potential acidity (H + Al), boron, copper, iron, manganese, zinc, sum of exchangeable bases, effective CEC, base saturation of CEC at pH 7.0 (V), and total carbon. For this purpose, composite samples were prepared from each experimental combination and respective replicate. Thus, the soil chemical characterization before the treatment's application to evaluate their initial conditions is presented in Table 2.

**TABLE 2:** Chemical characterization of the evaluated soils, before the application of the treatments.

Variables	Original soils		
	Oxisol	Inceptisol	Entisol quartzipsamment
Organic carbon, dag kg^−1^	3.40	2.14	<1.65
Organic matter, dag kg^−1^	5.86	3.69	<2.84
pH	4.50	5.00	5.40
Phosphorus, mg dm^−3^	3.45	4.95	4.20
Potassium, mg dm^−3^	45.00	43.00	25.00
Calcium, cmol_c_ dm^−3^	0.15	1.14	0.13
Magnesium, cmol_c_ dm^−3^	0.10	0.37	0.02
Aluminum, cmol_c_ dm^−3^	1.39	0.92	0.90
Potential acidity, cmol_c_ dm^−3^	10.90	5.80	2.20
Boron, mg dm^−3^	0.54	0.58	0.57
Copper, mg dm^−3^	1.14	0.98	0.73
Iron, mg dm^−3^	45.19	80.89	61.25
Manganese, mg dm^−3^	5.42	18.93	1.00
Zinc, mg dm^−3^	1.61	1.34	0.60
Sb, mg dm^−3^	0.37	1.62	0.21
Effective CEC, cmol_c_ dm^−3^	11.27	7.42	2.41
V, %	3.25	21.83	8.79
Total carbon, g dm^−3^	3.40	2.14	0.51

*Note*: Organic carbon: Colorimetric method; pH in water: electrochemical measurement; P, K: Mehlich‐I method; Ca, Mg, Al: Potassium chloride (KCl) Extractor 1 mol L^−1^; Potencial acidity: Extractor SMP; B: Barium Chloride Solution; Cu, Fe, Mn, Zn: Mehlich‐I extractor; sum of exchangeable bases (Sb): Ca + Mg + K; cation exchange capacity (CEC): Sb + Al.

After applying the treatments, soil fertility characterization was also carried out (Table 3).

**TABLE 3:** Chemical characterization of the evaluated soils after treated domestic wastewater (TWW) and artificial wastewater (AWW) miscible displacement tests.

Variables	TWW			AWW		
	Oxisol	Inceptisol	Entisol	Oxisol	Inceptisol	Entisol
Organic carbon, dag kg^−1^	2.09	<1.65	<1.65	2.27	<1.65	<1.65
Organic matter, dag kg^−1^	3.60	<2.84	<2.84	3.91	<2.84	<2.84
pH	5.60	5.30	5.40	5.60	5.30	6.10
Phosphorus, mg dm^−3^	3.11	4.49	66.70	2.42	3.34	2.19
Potassium, mg dm^−3^	64.00	67.00	78.00	50.00	53.00	69.00
Calcium, cmol_c_ dm^−3^	0.43	0.79	0.26	0.18	0.85	0.08
Magnesium, cmol_c_ dm^−3^	0.20	0.24	0.11	0.24	0.20	0.10
Aluminum, cmol_c_ dm^−3^	0.58	0.46	0.23	0.69	0.69	N/D
Potential acidity, cmol_c_ dm^−3^	10.40	6.80	1.60	8.40	4.50	1.20
Boron, mg dm^−3^	<0.10	0.11	0.11	0.10	0.10	<0.10
Copper, mg dm^−3^	2.53	1.26	0.27	2.73	1.50	0.25
Iron, mg dm^−3^	151.85	188.16	94.31	177.63	171.06	117.60
Manganese, mg dm^−3^	8.48	29.89	2.01	9.51	34.73	1.41
Zinc, mg dm^−3^	4.32	6.59	3.16	7.69	7.19	4.00
Sb, mg dm^−3^	0.79	1.20	0.57	0.55	1.19	0.36
Effective CEC, cmol_c_ dm^−3^	11.19	8.00	2.17	8.95	5.69	1.56
V, %	7.09	15.01	26.25	6.12	20.85	22.95
Total carbon, g dm^−3^	2.09	1.47	0.40	2.27	1.27	0.37

*Note*: Organic carbon: Colorimetric method; pH in water: electrochemical measurement; P, K: Mehlich‐I method; Ca, Mg, Al: Potassium chloride (KCl) Extractor 1 mol L^−1^; Potencial acidity: Extractor SMP; B: Barium Chloride Solution; Cu, Fe, Mn, Zn: Mehlich‐I extractor; sum of exchangeable bases (Sb): Ca + Mg + K; cation exchange capacity (CEC): Sb + Al.

### 2.2 Miscible displacement and breakthrough curve tests

The soil columns were saturated with deionized water by capillarity and leached intermittently to remove ions present in both solid and liquid phases of the soil, as well as those in the regions of stagnant flow (Cote et al., 2000). To verify the soil ion removal, the electrical conductivity of the effluent solution to the soil column was measured by potentiometry until it reached a maximum value of 0.02 dS m^−1^.

The miscible displacement assays were performed using Mariotte's bottles to maintain a constant hydraulic head on the soil columns. Both ends of the soil column were sealed with weldable PVC caps with a sealing ring. The caps had a hole in their center for connecting a flexible hose for leachate collection. Additionally, segments of geotextile fabric were placed at both ends of the columns to prevent the passage of solids. The entire experiment was conducted with an upward flow direction.

After confirming the steady‐state flow regime of deionized water through the soil column, the miscible displacement test was initiated with the respective water qualities for each soil type. The concentrations of solutes, both initial (*C*_0_) and effluent (C), were determined using a flame photometer and inductively coupled plasma optical emission spectrometry, following the methodologies present in the Standard Methods for the Examination of Water and Wastewater 3500 e 3120 (APHA; AWWA; WEF, 2022).

The aliquots were acidified with 1% nitric acid (v/v) for preservation and filtered through qualitative filter paper to retain oil and grease. Collection continued until the relative concentration (*C*/*C*_0_) of at least one of the elements presented in the effluent column reached 1. This measure was adopted due to the competition between the ions and the affinity between the adsorption sites of the soils and the solution ions, ensuring that the experiment duration remained feasible.

To determine the breakthrough curve, the solutions collected from the miscible displacement assay had their volume converted to pore volume, referring to the porosity of the soil contained in the column (Equation 4).

(4) $$V_{P}=\frac{V_{\mathrm{effluent}}}{V_{\mathrm{TP}}}$$

where *V*_P_ is the volume of pores of each pot (dimensionless), *V*_effluent_ is the volume of effluent collected (m^3^), and *V*_TP_ is the total volume of pores of the column (m^3^). The total pore volume of the column can be calculated by Equation (5).

(5) $$V_{\mathrm{TP}}=\alpha .V_{S}=\left(1-\frac{\rho_{s}}{\rho_{p}}\right).\pi \frac{d^{2}}{4}L$$

where *d* is the internal diameter (cm) and *L* is the height of the soil column (cm).

The transport parameters were obtained using STANMOD software, version 2.08 (Šimůnek et al., 2008), applying an inverse problem solution (CFITIM—“Code for determining nonequilibrium transport parameters from solute displacement experiments”). The parameters determined by the inverse problem were the retardation factor (*R*) and the Péclet number (Pe). From these, the linear partition coefficient of the soil solid phase (*K*_d_) for each evaluated ion (Equation 6) and the hydrodynamic dispersion coefficient (*D*) (Equation 7) were calculated.

(6) $$R=1+\frac{\rho_{s}K_{d}}{\alpha }$$

(7) $$\mathrm{Pe}=\frac{v.L}{D}$$

where *R* is the retardation factor (dimensionless), *K*_d_ is the partition coefficient of the soil solid phase (cm^3^ g^−1^), Pe is the Péclet number, *v* is the real velocity of water in the pores (cm min^−1^), and *D* is the hydrodynamic dispersion coefficient (cm^2^ min^−1^). It is important to note that there is no consensus on the values for the classification of the Pe (Huysmans & Dassargues, 2005). Therefore, the classification proposed by Manoel Filho (2008) was adopted.

The experiment was conducted in an entirely randomized design with three repetitions, totaling 18 soil columns (3 soil classes × 2 water qualities). To reduce data dimensionality, multivariate analyses of the observed data were performed to explain the relationships between the phenomena of retention and movement of solutes in the porous media as a function of the wastewater application. For this, the transport parameters R and D, mineralogy, and texture were considered, in addition to the variation of organic matter, CEC, potassium, calcium, and magnesium in soil. To this, a principal component analysis (PCA) and a hierarchical cluster analysis (HCA) were performed using RStudio software, using the packages “FactoMineR” (Le et al., 2008), “factoextra” (Kassambara & Mundt, 2020), and “stats” (R Core Team, 2022). The data were standardized using Equation (8) to assign the same weight to the variables, with a mean equal to one and a variance equal to zero (Fávero et al., 2009; Vicini et al., 2018; Ward, 1963).

(8) $$Z_{\mathrm{ij}}=\frac{X_{\mathrm{ij}}-{\bar{X}}_{j\;}}{S_{j}}$$

where *Z*_ij_ is the standardized variable of *X*_ij_, *X*_ij_ is the variable “*j*” referring to observation “*i*,” ${\bar{X}}_{j}$ is the arithmetic mean of the “*j*” variables, and *S_j_* is the sample standard deviation of “*j*” variables.

In the PCA, the main components related to the observed data were created, and the most relevant variables were evaluated (Alves et al., 2018). These principal components were obtained by the linear combination of the eigenvectors and the original standardized variables (Hardle & Simar, 2015). PCA was performed to identify the primary factors accounting for the variability among samples. This analysis was guided by the eigenvalues (>1) and the cumulative variance. For interpretation, we considered the loadings, which represent the contribution of each variable, and the scores, which allowed for the visualization of the sample distribution.

For HCA, the similarities between the soils and their retention properties of the evaluated ions were grouped. A dendrogram was built using the Ward agglomerative method and the Euclidean distance as a measure of similarity (Equation 9) (Vicini et al., 2018; Ward, 1963). Its interpretation was carried out to reveal patterns of similarity and differentiation among the groups. In this case, the smaller the distance, the greater the similarity between the observed data (Alves et al., 2018).

(9) $$d_{ik}=\sqrt{\sum_{j=1}^{p}{\left(Z_{ij}-Z_{kj}\right)}^{2}}$$

where *d_ik_* is the Euclidean distance between the standardized variables *Z_ij_* and *Z_kj_* (Vicini et al., 2018).

## 3 RESULTS AND DISCUSSION

### 3.1 Chemical characteristics of the soils and transport parameters of Na^+^, K^+^, Ca^2+^, and Mg^2+^

For the three types of soils studied and both wastewaters, except for Entisol Quartzipsamment and TWW, which remained constant at 5.4, an increase of approximately 15% in soil pH was observed, where values went to 5.6 for Oxisol, 5.3 for Inceptisol, and 6.1 for Entisol Quartzipsamment. This may have occurred due to the adsorption of non‐acidic cations such as Na^+^, K^+^, Ca^2+^, and Mg^2+^, which replace H^+^ ions, reducing acidity and increasing soil pH (Brady & Weil, 2013). Such an increase tends to promote the addition of negative charges in 1:1 type silicate clays, such as kaolinite, and iron and aluminum oxides, such as goethite and gibbsite, thereby increasing the CEC (Brady & Weil, 2013). Moreover, the presence of free hydroxyl ions (OH^−^), bicarbonates (HCO_3−_), and carbonates (CO_3_^2−^) in treated wastewater may react with the H^+^ ions in the soil, leading to an increase in soil pH (Hillel, 1998).

However, a reduction in CEC was observed for almost all soils, which was more pronounced for AWW. The CX for TWW was the only soil that had an increase in CEC. This may be related to the dispersion of clay due to the presence of Na^+^ in TWW and NaCl in AWW, as Na^+^ was the ion in the highest concentration, or it may be due to the substitution of bivalent cations, such as Ca^2+^ and Mg^2+^ – which were in lower concentration, by monovalent ones, such as Na^+^ and K^+^ (Meurer et al., 2017).

Another factor that may have contributed to the decrease of soil CEC is the adsorption of ions such as Cu and Zn, which can be adsorbed by the inner‐sphere complex, resulting in strong retention and a reduction in soil CEC (Brady & Weil, 2013; Meurer et al., 2017). In this context, an increase in Cu and Zn concentrations was observed for the soils after the application of both wastewater types (Table 3), except for Cu in Entisol Quartzipsamment, which showed a decrease. This may be linked to the low affinity of Entisol Quartzipsamment for Cu, as evidenced by Merlo et al. (2024, 2023). On the other hand, Entisol Quartzipsamment exhibited the highest increase in Zn concentration among the evaluated soils, approximately 358%.

Merlo et al. (2024) evaluated the retention capacity of Cu and Zn by the same soils evaluated in this study and verified that Entisol Quartzipsamment has a high affinity for Zn^2+^, quickly reaching the equilibrium concentration in a monocomponent system. The authors highlight that the high fixation of Zn by Entisol Quartzipsamment acts to reduce the mobility of this ion in the porous media and avoid groundwater contamination.

The increase in soil pH increases the availability of K^+^, Ca^2+^, and Mg^2+^ while reducing the availability of Al in tropical soils by precipitating available Al (Malavolta et al., 1997). The concentration of Al decreased in all soils and both wastewater types (Tables 2 and 3). Thus, it can be highlighted that the application of TWW and AWW to these soils promotes the removal of Al.

For the Oxisol, the Na^+^ for both water qualities was the first ion to reach a relative concentration equal to 1 during the tests, which occurred at an average pore volume of 4.3. The lowest *R*_Na_ and *D*_Na_ (Table 4) were also observed compared to the other ions studied, evidencing the Na^+^ low affinity for the available adsorption sites of Oxisol and its low dispersity. This can be explained by its monovalent nature, relatively large hydrated ion radius, and the predominance of convection transport. It is also noteworthy that Oxisol showed the highest *K*_dNa_ among the soils, despite having a low affinity for this ion. This may be related to the higher content of organic matter and CEC due to the presence of kaolinite, hematite, gibbsite, goethite, and magnetite, which, despite being minerals with low CEC (Ronquim, 2010; Brady & Weil, 2013), contributed to the retention of ions in Oxisol.

**TABLE 4:** Mean and standard deviation of the retardation factor (R), dispersion‐diffusion coefficient (D), partition coefficient of the soil solid phase, and Péclet number for each evaluated ion, soil, and water quality.

		R (dimensionless)		D (cm^2^ h^−1^)		*K*_d_ (cm^3^ g^−1^)		Pe (dimensionless)	
Soil and water quality	Ion	Mean	Standard deviation	Mean	Standard deviation	Mean	Standard deviation	Mean	Standard deviation
O_TWW_	Na	2.15	0.42	6.42	5.15	0.69	0.25	3.64	2.08
	K	41.76	41.53	209.94	284.70	24.60	25.07	1.20	1.11
	Ca	248.18	58.80	534.65	33.56	149.17	35.49	0.03	0.01
	Mg	239.92	76.33	189.02	120.84	144.19	46.06	0.09	0.03
O_AWW_	Na	3.93	0.38	39.50	16.22	1.77	0.23	1.19	0.57
	K	132.02	30.38	627.46	326.89	79.07	18.33	0.08	0.03
	Ca	85.15	59.24	657.92	446.06	50.78	35.75	0.27	0.33
	Mg	169.90	30.81	544.89	129.46	101.93	18.59	0.07	0.02
I_TWW_	Na	1.86	0.04	0.73	0.37	0.42	0.02	4.77	1.28
	K	10.87	3.24	4.17	2.38	4.84	1.59	0.88	0.26
	Ca	1.50	1.47	0.77	0.70	0.24	0.72	9.10	5.54
	Mg	82.47	3.37	116.92	105.88	39.96	1.65	0.04	0.02
I_AWW_	Na	1.65	0.30	4.14	3.66	0.32	0.15	3.67	2.12
	K	113.46	53.14	174.04	94.71	55.17	26.07	0.06	0.03
	Ca	49.68	39.88	63.42	51.59	23.88	19.57	17.49	14.20
	Mg	37.20	32.27	436.18	312.31	17.76	15.83	338.19	478.24
E_TWW_	Na	2.58	0.15	0.07	0.01	0.35	0.03	32.37	8.44
	K	142.70	188.65	7.25	8.39	31.00	41.27	1.87	1.85
	Ca	127.22	98.41	58.85	44.25	27.62	21.53	0.13	0.14
	Mg	204.03	115.79	22.94	6.24	44.42	25.33	0.11	0.04
E_AWW_	Na	3.66	0.93	1.42	0.85	0.58	0.20	2.86	1.27
	K	499.41	210.71	38.66	8.51	109.05	46.10	0.08	0.01
	Ca	4.62	5.33	11.65	16.23	0.79	1.17	259.23	360.41
	Mg	245.98	103.37	43.94	14.51	53.60	22.62	0.07	0.01

*Note*: E: Entisol Quartzipsamment; I: Inceptisol; O: Oxisol; AWW: artificial wastewater; TWW: treated domestic wastewater.

Abbreviation: kd, linear partition coefficient of the soil solid phase.

Barbosa et al. (2022) evaluated the effect of treated wastewater fertigation in an Oxisol over 4 years in an area cultivated with forage grass, located in a humid tropical region. The authors observed that, despite the high concentration of sodium present in the treated wastewater, the adsorption rate was low and did not cause salinization and sodification of the soil. This corroborates the low affinity observed by the three soils studied and the high sodium mobility noted in this study.

On the other hand, for the last pore volume collected for Oxisol (12.3 pore volume), the relative concentration of Ca^2+^ was 60% for TWW. This affinity can be observed by the higher values for *R*_Ca_ and *K*_dCa_ (Table 4). For AWW, Mg^2+^ showed the greatest affinity with the Oxisol, reaching only 30% of its relative concentration, and showed higher *K*_dMg_ and *R*_Mg_ values (Table 4). In both cases, high *D*_Ca_ and *D*_Mg_ (Table 4) were observed, implying a greater mixture of these ions in the solution in the Oxisol, due to its higher average pore water velocity (Carmo et al., 2010). Thus, despite the high dispersion observed, the affinity of Ca^2+^ and Mg^2+^ for the porous media exchange complex, combined with their low concentration (4.0 and 6.2 mg/L, in that order) and high binding energy, because they are bivalent cations, resulted in ions retained with the advance of the contamination front through the soil pores. Thus, at the end of the experiment, despite the dispersion, the relative concentration observed was low.

It was observed that the real pore water velocities for TWW (Oxisol: 43.56 cm h^−1^, Inceptisol: 9.31 cm h^−1^, and Entisol Quartzipsamment: 6.69 cm h^−1^) were lower than those for AWW (Oxisol: 114.46 cm h^−1^, Inceptisol: 24.72 cm h^−1^, and Entisol Quartzipsamment: 9.18 cm h^−1^). Consequently, the D for TWW, except for Ca^2+^ in Entisol Quartzipsamment, was also lower. This may have occurred due to the presence of solids in the wastewater, which can clog the soil pores. In addition, the higher ionic concentration can lead to a more intense interaction between ions, forming ionic associations and reducing their mobility (Bešter‐Rogač et al., 2016). Thus, a more concentrated solution of ions, such as sewage, has higher viscosity relative to AWW, slowing down the movement of ions through the porous media, which can also reduce the hydrodynamic dispersion coefficient (Li & Chang, 1955).

Entisol Quartzipsamment exhibited a strong affinity for K^+^, especially for AWW. In this study, the highest values of *R*_K_ and *K*_dK_ were observed among the soils and ions, resulting in a higher concentration of K^+^ in the soil after the tests (Table 4). Similarly, for TWW, both K^+^ and Mg^2+^ showed high R and *K*_d_, which contributed to the low dispersion of ions in the Entisol Quartzipsamment. Additionally, among the soils for each of the evaluated ions, it was observed that the lowest *D* values were found for the Entisol Quartzipsamment (Table 4). This is also related to the lower porosity of Entisol Quartzipsamment compared to the other soils, which hinders the dispersion of ions and limits their movement. It can also be highlighted that the affinity and retention of K^+^ by Entisol Quartzipsamment contributed to reducing its dispersion (*D*_K_).

### 3.2 Multivariate analysis for the application of TWW and AWW

By multivariate analysis, both for the application of TWW and AWW, the first two principal components explained 100% of the data variance. The first principal component (PC1) explained 60.82% of the variability for TWW and 64.67% for AWW (Table 5).

**TABLE 5:** Correlation between the transport parameters and the physical, chemical, and mineralogical attributes of the soils.

	PC1	PC2
Eigenvalue	13.274	5.487
Variability (%)	57.715	23.858
Cumulative (%)	57.715	81.572
	Correlations	
*R*_Na_	−0.105	**0.775**
*R*_K_	−0.655	0.418
*R*_Ca_	0.436	0.480
*R*_Mg_	−0.224	**0.891**
*D*_Na_	0.648	0.442
*D*_K_	**0.744**	0.417
*D*_Ca_	**0.808**	0.586
*D*_Mg_	**0.744**	−0.044
ΔOM	−**0.984**	0.069
ΔCEC	0.351	−0.351
ΔK	−**0.915**	0.315
ΔCa	0.316	0.495
ΔMg	−**0.748**	0.646
Kaolinte	**0.766**	−0.637
Hematite	**0.809**	0.577
Gibbsite	**0.996**	−0.034
Goethite	**0.964**	0.249
Magnetite	**0.809**	0.577
Quartz	−**0.996**	−0.003
Muscovite	−**0.908**	0.411
Clay	**0.967**	0.238
Silt	0.517	−**0.850**
Sand	−**0.995**	−0.041

*Note*: *R*_Na_, *R*_K_, *R*_Ca_, *R*_Mg_: retardation factor for sodium, potassium, calcium, and magnesium ions; *D*_Na_, *D*_K_, *D*_Ca_, *D*_Mg_: hydrodynamic dispersion coefficient for sodium, potassium, calcium and magnesium ions; ΔOM and ΔCEC: variation of organic matter and CEC; ΔK, ΔCa and ΔMg: variation in the concentration of potassium, calcium, magnesium and ions present in soils. Bold values indicate strong correlation (0.75 > $Z_{ij}$ > 1.0) according to Vieira (2018).

Abbreviation: PC, principal component.

PC1 was positively correlated to *D* for all ions evaluated, these correlations being strong for *D*_K_, *D*_Ca,_ and *D*_Mg_; kaolinite, hematite, gibbsite, goethite, magnetite, and clay. Meanwhile, it was strongly and negatively correlated to variations in organic matter, potassium, and magnesium concentration in the soil, as well as quartz, muscovite, and sand.

**FIGURE 2:**
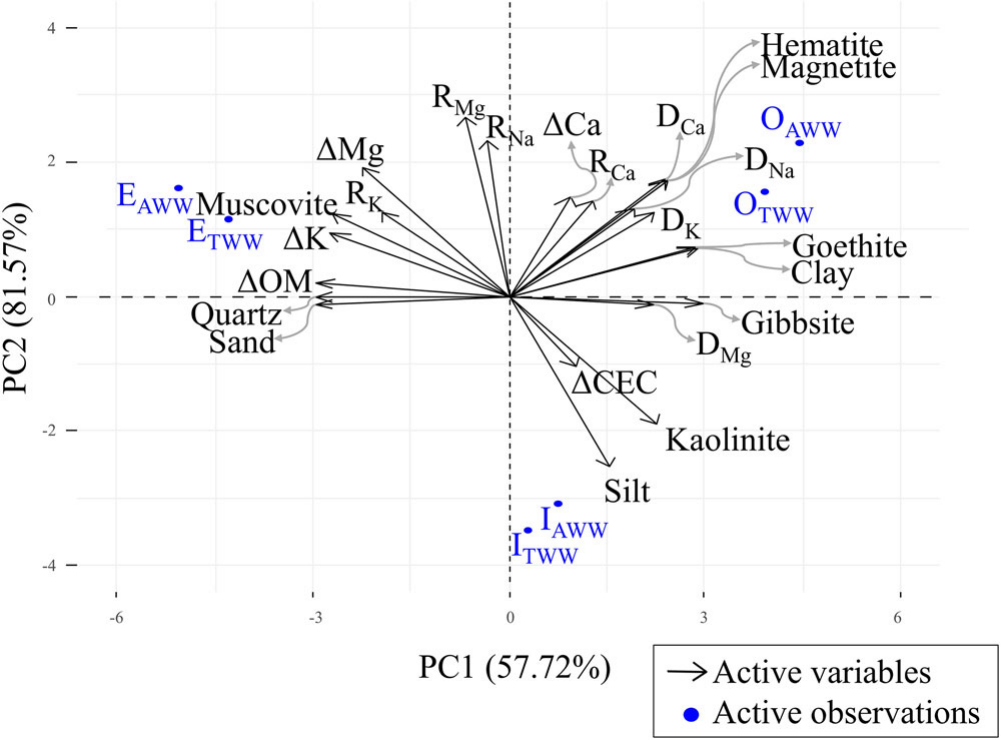
Biplot of the principal components (PC1 and PC2) of the variables studied for treated domestic wastewater (TWW) and artificial wastewater (AWW). E: Entisol Quartzipsamment; I: Inceptisol; O: Oxisol. CEC, cation exchange capacity

The second principal component (PC2) was strongly and positively correlated with *R*_Na_ and *R*_Mg_. On the other hand, PC2 was strongly and negatively related to silt. The further variables were correlated differently with the principal components, as seen in Figure 2.

It was found that PC1 overall represents the dispersion‐diffusion of ion behavior. Additionally, PC1 is also explained by the clay fraction and the minerals kaolinite, hematite, gibbsite, goethite, and magnetite, while PC2 reflects the ions' movement retardation, except for Ca^2+^, given the absence of a negative correlation between *R*_Ca_ and PC1. It is also worth noting that ΔK and ΔMg, although not directly related to PC2, are positioned in the same quadrant of the previously mentioned variables and, therefore, corroborate the explanation of PC2. Neither retention nor soil Ca variation was strongly related to either PC1 or PC2.

Convective transport of the Na^+^ was observed, except for the Entisol Quartzipsamment for TWW, where its transport occurred by mechanical dispersion. In this case, the Na^+^ ions diluted in the wastewater were carried along with the movement of the wetting front. For K^+^ and Mg^2+^, diffusion transport was observed in most soils. For Ca^2+^, both convection transport and mechanical dispersion and diffusion were observed, varying with the soil type.

It is important to highlight that the use of TWW and AWW led to similar results regarding ion movement and retention, as can be observed by the location of the cases (soils and solutions) in the PCA quadrants (Figure 2). Furthermore, most transport parameters showed the same contribution behavior to PC1 and PC2 (Table 5), with the only exception being *R*_Ca_, as previously highlighted (Table 5; Figure 2). This demonstrates the applicability of the AWW as a predictor of domestic wastewater Na, K, and Mg ion retention behavior in tropical soils, even with simpler chemical characteristics. From a practical standpoint, artificial solutions are easily produced, and they represent a minor risk of contamination for humans and the environment.

**FIGURE 3:**
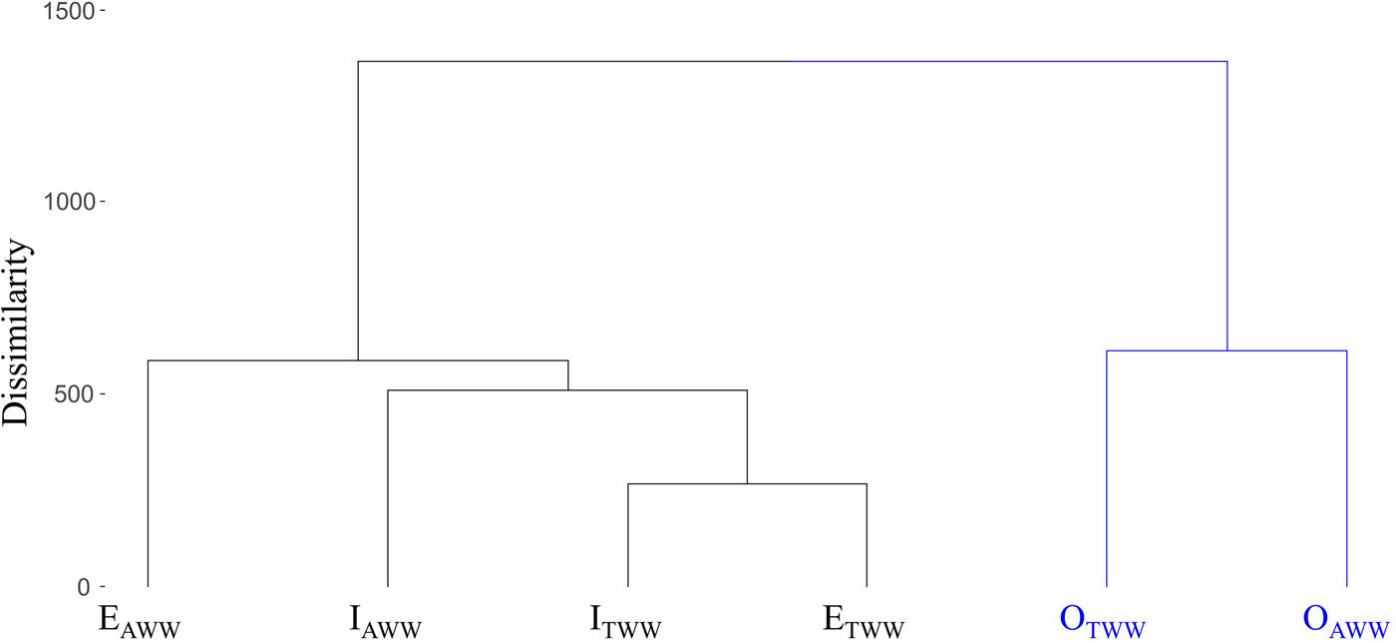
Analysis of hierarchical soil clustering for treated domestic wastewater (TWW) and artificial wastewater (AWW). E: Entisol Quartzipsamment; I: Inceptisol; O: Oxisol.

Based on the physical, chemical, and mineralogical properties of the soils, the dendrogram was cut to obtain two groups (1000 height), as shown in Figure 3.

The hierarchical clustering illustrates the disparity in Oxisol ion retention capacity compared to Entisol Quartzipsamment and Inceptisol, based on the lower CEC and organic matter content of the latter (Table 3).

Both Oxisol and Entisol Quartzipsamment contributed to PC1 (Figure 2); however, Oxisol has shown a positive relation, while Entisol Quartzipsamment exhibited a negative relation, highlighting the antagonism between them regarding ion retention capacity. This retention capacity is also evident from fertility analysis (Table 3), in which Oxisol exhibits CEC approximately 80% higher than that of Entisol Quartzipsamment. In addition, Oxisol's mineralogical composition (Figure 1) includes 30.3% of kaolinite, a 1:1 clay that, despite its low CEC, contributes to the soil's retention capacity, along with the organic matter. Entisol Quartzipsamment, however, has 89.6% quartz, a mineral with extremely low or nonexistent CEC (Melo et al., 2019).

Inceptisol showed an inverse relationship with PC2 (Figure 2), since this soil presented the lowest value for the retardation factor for all ions, except for Ca^2+^ in AWW. This indicates that there was a lower delay in the ions’ movement relative to the velocity of the contamination front, as well as a low affinity for the adsorption sites available in the Inceptisol, evidenced by the lower partition coefficient of the solid phase compared to the other soils.

Thus, the negative relation between Entisol Quartzipsamment and Inceptisol with the principal components, the lower affinity for the evaluated ions, the lower retardation factor, the lower real pore water velocity, and the lower dispersion of the solutes also contributed to grouping these soils (Figure 3). According to Merlo et al. (2024, 2023), Oxisol is characterized by its high saturated hydraulic conductivity and can behave similarly to sandy soils with high porosity, allowing metals to leach and contamination of the groundwater. However, in this study, Oxisol generally exhibited a high affinity for the evaluated cations, resulting in greater retention and retardation, particularly for Ca^2+^ and Mg^2+^.

Another factor that can be highlighted is the depth of the Oxisol profile, which, due to the long path that the contamination front travels, contributes to its attenuation and the preservation of groundwater quality. Therefore, it is evident that the application of TWW and AWW in Oxisol can be beneficial from the agricultural and environmental perspectives, due to the increase of K^+^, Ca^2+^, Mg^2+^, Cu^2+^, Fe^2+^, Mn^2+^, Zn^2+^, and the sum of bases. Barbosa et al. (2022) also verified an increase in the fertility of an Oxisol after application of treated wastewater and concluded that fertigation of clayey soils, located in humid tropical regions, cultivated with forage grasses, is recommended.

Similar to Oxisol, Entisol Quartzipsamment showed an increase in K^+^, Ca^2+^, Mg^2+^, Fe^2+^, Mn^2+^, Zn^2+^, and sum of bases for both wastewaters. Despite its sandy texture, the low porosity of this soil may contribute to reduced dispersion of solutes, thereby attenuating potential contamination.

Thus, a TWW with similar quality as the one of this study, which resembles common quality domestic sewage but diluted, can be used as a viable source of water and nutrients in Oxisol and Entisol Quartzipsamment, especially in places where freshwater availability for irrigation is limited. Furthermore, supplying nutrients to the soil using TWW can reduce the reliance on chemical fertilizers, leading to cost savings for farmers, while also contributing to nutrient cycling and reducing water pollution. However, it is essential to monitor the quality of the wastewater used and regularly assess the physical and chemical properties of the soils to prevent salinization and metal accumulation and to ensure the preservation of food security.

To mitigate the adverse effects of Na^+^, several practical management strategies can be adopted. The application of gypsum (CaSO_4_·2H_2_O) is one of the most effective practices, particularly when the wastewater exhibits high SAR and EC values that could lead to soil dispersion, such as the wastewater used in this study (Table 1). The calcium provided by the gypsum replaces the sodium on the cation exchange complex, which in turn reduces the risk of soil dispersion. Additionally, complementary practices like proper irrigation scheduling are crucial to prevent excessive leaching and soil salinization.

Regarding the TWW application, it was found that the most suitable soils are Oxisol and Entisol Quartzipsamment. Nevertheless, for practical agricultural use, Oxisol proves to be a better choice for TWW application and plant cultivation. This is attributed to its higher fertility, its strong affinity for the ions analyzed, and its greater profile depth, which promotes better root growth and development.

## 4 CONCLUSION

The application of TWW and AWW resulted in an increase in soil pH and a reduction in acidity, along with enhanced availability of K^+^, Ca^2+^, and Mg^2+^ in tropical soils, a fact of relevant interest from an agricultural perspective. However, a reduction in the CEC of the soils was observed, with a more pronounced decrease for AWW.

Oxisol showed higher affinity for the ions evaluated, except for K^+^, which had higher affinity for Entisol Quartzipsamment. These soils also showed the greatest delays in ion transport in relation to the wetting front, which is important due to the availability of exchangeable ions for plants. On the other hand, Inceptisol, in general, had the lowest retardation factors and lowest affinity with ions, which would result in greater leaching of ions and greater possibility of groundwater contamination.

It is important to emphasize that the presence of sodium in TWW is a point of attention, as it was observed to have a low affinity for the soils and exhibited the lowest retardation factor among the evaluated elements. In this way, sodium can move easily through the soil profile, and, depending on its concentration, it can disperse the clay, reduce the CEC, cause salinization, and degrade the soil structure.

## AUTHOR CONTRIBUTIONS

**Marina N. Merlo**: Data curation; formal analysis; investigation; methodology; writing—original draft. **Michael S. Thebaldi**: Conceptualization; formal analysis; funding acquisition; investigation; methodology; project administration; resources; supervision; writing—review and editing. **Miguel A. C. Alvarez**: Investigation. **Daniela C. de Jesus**: Investigation; writing—original draft. **Jaqueline dos S. Soares**: Investigation. **Elvis M. de C. Lima**: Funding acquisition; supervision. **Mateus A. da Silva**: Investigation. **Luiz A. Lima**: Funding acquisition; supervision. **Luiz F. C. de Oliveira**: Funding acquisition; supervision.

## CONFLICT OF INTEREST STATEMENT

The authors declare no conflicts of interest.
